# A dual role for adeno-associated virus in human health

**DOI:** 10.1186/s12985-023-02196-8

**Published:** 2023-10-10

**Authors:** Natalia M Araujo

**Affiliations:** Laboratory of Molecular Virology and Parasitology, Oswaldo Cruz Institute, FIOCRUZ, Rio de Janeiro, RJ Brazil

**Keywords:** AAV, Hepatocellular carcinoma, Hepatitis, Children, Gene therapy

## Abstract

Adeno-associated virus (AAV) differs from most other viruses, as it requires the simultaneous presence of a helper virus for an active infection. Up to 80% of the human population is seropositive for AAV antibodies. AAV has been known to be a non-pathogenic virus and an inhibitor of carcinogenesis caused by coinfecting viruses. However, the recent reports associating AAV infection with hepatocellular carcinoma development and the mysterious cases of acute severe hepatitis in children have challenged the idea that AAV is a harmless virus. Herein, we explore the usefulness of AAV in gene therapy and the importance of AAV as a protector or perpetrator in human carcinogenesis, ultimately reflecting on the dual role of AAV in human health.

## Main text

Discovered as an adenovirus stock contaminant in the mid-1960s, adeno-associated virus (AAV) is a non-enveloped mono-stranded DNA virus that belongs to the *Dependoparvovirus* genus within the *Parvoviridae* family [1]. AAV infects a variety of animal species, including humans, and has a global seroprevalence that ranges from around 30–80% in the human population, depending on the AAV serotype and analyzed cohort [2, 3]. The liver has been shown to be the main infection site of AAV, with the bone marrow, spleen and uterus being important secondary infection sites [4]. Unlike most other viruses, AAV requires the simultaneous presence of a helper virus to undertake active infection [5].

The most visible and popular application of AAV is in gene therapy, where it offers the unique capacity of recombinant AAV (rAAV) vectors to transduce dividing and non-dividing cells with high efficiency to yield long-term transgene expression, relatively low immunogenicity, and selective tissue tropism [6]. Gene therapy has wide-ranging potential to improve the treatment options for patients suffering from inherited or acquired diseases and can pave the way for a new era in the treatment of uncommon illnesses and cancer [7], as well as in the prevention of infectious diseases [8]. Some AAV-based gene therapy products have already been authorized for human use, including Glybera (lipoprotein lipase deficiency), Luxturna (retinal dystrophy), Zolgensma (spinal muscular atrophy), Hemgenix (hemophilia type B), Roctavian (hemophilia type A), and Upstaza (AADC deficiency) [9, 10].

Given that AAV is endemic in the human population, it is remarkable that many questions on its natural infection remain unresolved. There have been few research addressing the influence of AAV infection on human health, and the findings are inconsistent (reviewed in [11]). In general, AAV has been known to be a non-pathogenic virus and an inhibitor of carcinogenesis caused by coinfecting viruses. It was found to induce selective apoptosis in cells lacking active p53, inhibit tumor growth in mice [12], and suppress HPV-induced cell transformation in vitro and in vivo [13–15]. Consistent with this, epidemiological data suggest that AAV infection plays a tumor-suppressing role in HPV-related cervical cancer [16–18].

Surprisingly, the idea that AAV is a non-pathogenic virus has been challenged by recent reports. These studies found that clonal wild-type AAV (wtAAV) insertions in human cancer-driver genes were associated with rare cases of hepatocellular carcinoma (HCC) in a non-cirrhotic liver background [19–21]. In particular, La Bella and colleagues (2020) [19] provided a comprehensive examination of the wtAAV infection in the liver with a description of viral genotypes, molecular forms, helper virus relationship, and viral integrations. However, the magnitude of the genotoxic potential of wtAAV is still little known, considering the prevalence of natural infections and the few AAV-related HCC cases reported so far. Noteworthy, natural infection with AAV has no correlation with the delivery of current rAAV vectors for gene therapy, since a considerable portion of the wtAAV genome is deleted when developing AAV-based vectors, thus preventing rAAV from replicating. Despite the two independent mouse models of AAV vectorization that developed HCC by insertional mutagenesis [22, 23], the risk of rAAV-mediated oncogenesis in humans is theoretical since no confirmed genotoxic events have been documented to date [24].

Also surprising were the three independent studies published in the March 2023 issue of *Nature* demonstrating that infection with AAV serotype 2 (AAV2) was linked to recent clusters of unexplained acute severe hepatitis in children [25–27]. The mysterious cases of non-A to E hepatitis were first identified in April 2022 in Scotland and, by July 2022, the World Health Organization had reported over 1,000 probable cases across 35 countries, including 46 cases that required liver transplants and 22 deaths [28]. Based on metagenomic sequencing of available whole blood, plasma, stool, or liver tissue, the three investigative groups observed AAV2 positivity in 96%, 93%, and 81% of cases but only 9.2%, 3.5%, and 7% of controls in London [26], the U.S. [27], and Scotland [25], respectively. Many of the patients also showed evidence of infection with a helper virus, such as human adenovirus or herpesviruses. Furthermore, it is known that human leukocyte antigen (HLA) polymorphisms can affect both the susceptibility and the severity of viral infections, since this complex contains the key immune response genes determining peptide presentation to T cells [29]. Interestingly, genotyping analysis revealed that the affected children had a high frequency of HLA class II DRB1*04:01 allele (93% of cases compared to 16% of the overall United Kingdom population), supporting an immunological predisposition [25]. Consistent with this, liver samples from cases were found to be enriched in adaptive immune cells and immune-related proteins, and RNA transcriptome analysis of these samples pointed to the occurrence of active AAV2 infection [25, 26]. These data suggest that acute hepatitis was caused by aberrant immune responses, with AAV2 acting as a potential trigger for immune-mediated liver damage rather than as a hepatotoxic factor. Remarkably, the timing of the hepatitis outbreak coincided with the global relaxation of COVID-19 restrictions. Given that community lockdowns may have altered children’s regular exposure patterns and immunity, those with a specific genetic background may have been more susceptible to viral coinfections [30, 31]. Of note, three months after the publication of the *Nature* studies, Gates and colleagues (2023) [32] investigated the epidemiology of AAV in the United Kingdom by analyzing 300 pediatric respiratory samples collected before (April 2009–April 2013) and during (April 2022) the COVID-19 pandemic. Wastewater samples were also collected from 50 locations in London (August 2021–March 2022). Interestingly, the detection frequency of AAV2 was a sevenfold higher in the pediatric samples from 2022 than those from 2009 to 2013 (10% vs. 1.4%). Moreover, in wastewater collected in 2021, AAV2 sequences were either extremely low or absent, but increased in January 2022 and peaked in March 2022. Taken together, these findings support the etiological link between AAV2 infection and the cases of acute hepatitis in children, and demonstrate the potential of AAV2 to cause severe disease under specific conditions (e.g. host genetic background and increased postpandemic host susceptibility).

## Conclusion

Clearly, the recent studies associating AAV infection with HCC development and acute hepatitis in children are paving the way for renewed interest in wtAAV biology. Further research is needed to determine the impact of AAV infection and the frequency of insertional mutagenesis in additional cohorts of patients. Moreover, to evaluate whether AAV2 infection can cause, or contribute to, acute hepatitis in children, prospective and well-controlled follow-up studies are necessary. The existing literature on AAV shows us that the virus-host relationship is complex and the viruses can be useful in several healthcare contexts.

## Data Availability

Not applicable.
